# NanoShaperWeb: Molecular Surface and Pocket Detection Made Visual

**DOI:** 10.1021/acs.jcim.5c00821

**Published:** 2025-06-30

**Authors:** Carlo Abate, Eleonora Serra, Walter Rocchia, Andrea Cavalli, Sergio Decherchi

**Affiliations:** † Department of Pharmacy and Biotechnology (FaBiT), Alma Mater Studiorum - University of Bologna, via Belmeloro 6, 40126 Bologna, Italy; ‡ Computational & Chemical Biology, 121451 Fondazione Istituto Italiano di Tecnologia, via Morego 30, 16163 Genoa, Italy; ¶ Concept Lab, Fondazione Istituto Italiano di Tecnologia, via Morego 30, 16163 Genoa, Italy; § Centre Européen de Calcul Atomique et Moléculaire (CECAM), Ecole Polytechnique Fédérale de Lausanne, 1015 Lausanne, Switzerland; ∥ Data Science and Computation Facility, Fondazione Istituto Italiano di Tecnologia, via Morego 30, 16163 Genoa, Italy

## Abstract

Analyzing molecular surfaces to predict functional sites and identify protein cavities for small molecule binding is essential in structural biology and drug discovery, particularly when targeting allosteric sites or designing PROTACs. Moreover, measuring properties like volume, surface area, and pockets’ chemical descriptors helps in understanding protein function and improving drug development. Over the past decades, numerous surface and pocket-detection tools have been developed. While these tools provide valuable insights, they often require extensive postprocessing of text output files, making the analysis workflow cumbersome. To address this limitation, we introduce NanoShaperWeb, a web server that not only provides the computational capabilities of NanoShaper but also eliminates the need for manual text file processing through an intuitive web-based interface. Molecular surface and pocket-detection computations are performed remotely via a queue, with results visualized interactively and available for download. The application also delivers for each pocket DrugPred descriptors, enabling deeper insights into pocket features. By streamlining molecular analysis, this tool offers an efficient and accessible platform for researchers, supporting key stages of the drug design pipeline. The NanoShaperWeb tool is freely accessible online at https://nanoshaperweb.iit.it/ with no required registration.

## Graphical abstract

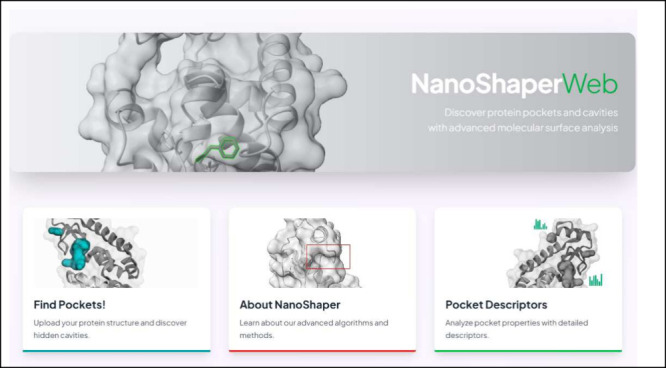

## Introduction

The drug discovery process is an intricate and time-consuming process^1,2^ spanning from understanding the underlying biology of the disease^3^ to the precise optimization of lead candidates, frequently leveraging computational methods.^4^ Over the past decades, *in-silico* approaches have played a transformative role in this process,^5^ particularly for analyzing molecular surfaces and predicting protein crevices suitable for accommodating therapeutic molecules. Among all biological interacting partners, proteins play crucial roles in various cellular processes, making them central to pharmacology.^6^

In this framework, a software capable of triangulating molecular surfaces and detecting protein cavities and pockets is crucial. Numerous pocket identification algorithms are available today and in this realm NanoShaper^7^ is a reliable and fast tool for analyzing molecular surfaces and detecting protein cavities and pockets. What makes it unique is its analytical treatment of the molecular surface (accuracy) and its multicore parallel code (speed). In this application note, we present NanoShaperWeb, a browser application built over the existing NanoShaper tool to expose its capabilities through a simple interface. Additionally, it provides DrugPred descriptors^8^ offering both geometric and chemical insights into detected pockets, providing a starting point for machine learning campaigns. The application can be freely accessed without registration at https://nanoshaperweb.iit.it/.

## Implementation

The web application is organized in a microservices architecture with three main components: the NanoShaper executable, a Flask-based Web Server, and the Browser App. Flask handles the back-end processing of requests and serving responses, while the Browser App provides an interactive user interface. All components are containerized using Docker to ensure consistent deployment and scalability. A high-level overview of the system architecture is presented in Figure 1.

**Figure 1:**
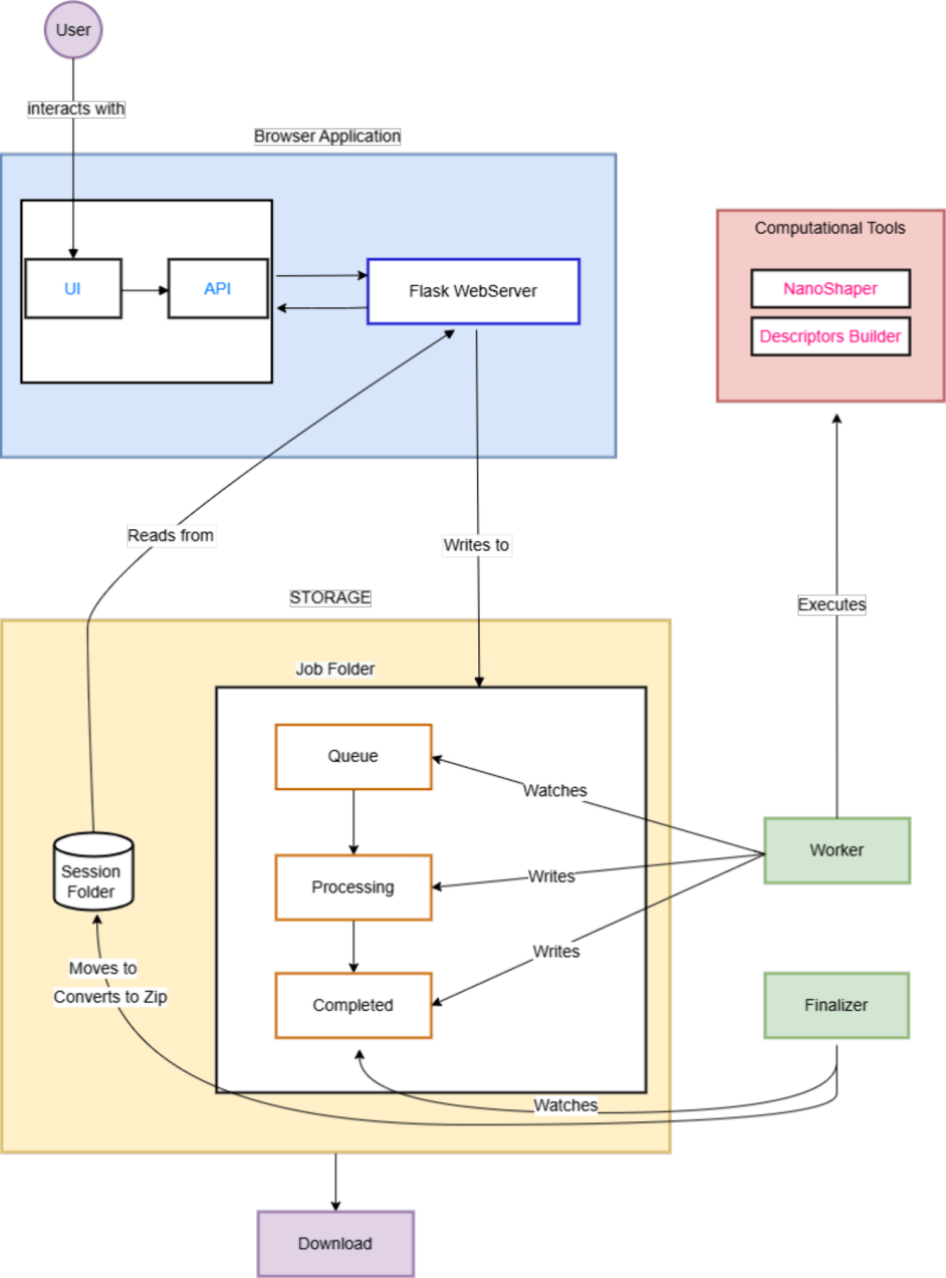
Diagram illustrating the architecture of NanoShaperWeb, outlining the interactions between the user, browser application, storage system, computational tools, and worker processes. The user interacts with the web interface, which communicates with a Flask-based web server. Submitted jobs are stored in the job folder and processed through a queue system. Workers execute computational tasks, interacting with computational tools such as NanoShaper and Descriptors Builder. Upon completion, results are finalized and stored, allowing users to download processed data.

### NanoShaper and Descriptors

The application is built around two NanoShaper functionalities, namely surface computation and pocket detection. The application provides visualization for all the results computed by NanoShaper, along with features such as the computation of the extended DrugPred descriptors we recently employed.^9^

#### Surfaces

NanoShaper drives computation of molecular surfaces by multiple definitions,^10,11^ including van der Waals (VdW), Connolly-Richards or solvent-excluded surface (SES), skin,^12^ and blobby.^13^ All these surfaces are automatically computed in the interface by running several times NanoShaper.

#### Pocket Detection

a pocket is a region of space accessible to a water molecule but inaccessible to a larger spherical probe. The detection is performed by computing the volumetric difference between two SESs with different probe radii, followed by cleanup and flood-filling algorithms to isolate individual pockets. NanoShaper can also identify closed cavities within the analyzed surface and provides an estimate of their volume.

#### Descriptors Builder

this is an in-house postprocessing tool,^9^ separate from NanoShaper, that runs after its analysis is completed. It characterizes the detected pockets using DrugPred-based geometric and chemical descriptors, as well as entrance area and entrance points.^9^ These include hydrogen-bond donor and acceptor surface areas, hydrophobic surface areas, compactness, and amino acid composition. The compactness descriptor quantifies how closely a pocket’s shape approximates a sphere, with values approaching 1 indicating more spherical geometry. The complete list of calculated descriptors is available at the Web site and in the Supporting Information.

### Web Server

The server is built using Flask, a lightweight web framework for Python. The server is responsible for handling HTTP requests, managing user sessions, processing PDB files, and orchestrating job execution. The backend architecture follows a modular design with distinct components:

#### API Layer

implements RESTful end points for handling client requests, organized into route modules for different functionalities (sessions, jobs, PDB processing, results). Each route module provides specialized end points that handle specific aspects of the application workflow.

#### Session Management

maintains user sessions and provides session-specific data storage without requiring user authentication. Session data is persisted to the file system for reliability and to allow users to resume their work across browser sessions using a unique session ID.

#### Job Queue System

implements a FIFO (First-In-First-Out) queue for processing requests of NanoShaper runs. Jobs move through multiple stages (incoming, queue, processing, completed, failed) with locking mechanisms to handle concurrent access. The system uses a file-system-based queueing for reliability and persistence across server restarts.

#### Worker Process

handles the actual execution of NanoShaper commands and postprocessing of results. The worker process operates independently from the web server, processing jobs sequentially from the queue. For improved throughput, multiple worker processes can run in parallel, each handling different jobs.

#### Finalizer Process

monitors completed runs and performs finalization tasks such as generating descriptors, creating pocket entrances, and preparing data for visualization. This process runs asynchronously to avoid blocking the worker and web server.

The server extensively uses the ProDy library^14,15^ for molecular structure manipulation and preprocessing. This includes parsing PDB files using parsePDB for loading structures, applying atom subselections with VMD-like syntax (e.g., “protein and not hetero”), removing duplicated atoms, and converting to the .xyzr format. The server applies Amber naming conventions via force field parameter files to ensure consistent atom naming across different inputs. For improved performance in computationally intensive tasks, several routines are implemented in C.

### Browser App

The client app, running in the browser, consists of components that provide the user interface and interact with the web server through asynchronous HTTP requests. Here we briefly report the employed frameworks and components.

#### Frontend Framework

uses HTML5, CSS with TailwindCSS for styling, and vanilla JavaScript for interactivity. The user interface is organized into sections for input configuration, job management, and result visualization.

#### 3D Visualization

employs 3DMol.js^16^ for molecular visualization, with custom wrapper classes to handle surface rendering, pocket highlighting, and interactive exploration. The visualization component supports multiple rendering modes, custom coloring of pockets, and interactive selection.

#### State Management

maintains visualization settings, selected pockets, and UI preferences on the server file system, with client-side synchronization for real-time updates. Session data including viewer state is persisted in JSON files on the server, allowing users to resume their visual analysis across different sessions. This server-side state management ensures consistent user experience throughout the application workflow.

#### Web Workers Architecture

implements a multithreaded client-side processing system using dynamically generated web workers for performance-critical tasks. These web workers handle resource-intensive operations such as downloading large result files while providing progress tracking, ensuring a responsive user experience without blocking the main UI thread. This approach maintains smooth interaction during computationally intensive processes and includes graceful fallback mechanisms when web workers are unavailable.

## Web Server Interface

The landing page consists of a hero banner with a bottom panel providing three links to individual pages: *Find Pockets*, *About NanoShaper* and *Pocket Descriptors*.

The *Find Pockets* page is the main interface where users can analyze protein structures and detect pockets; the *About* page gives a short description of the methods, while the *Descriptors* page provides a short explanation of the DrugPred descriptors^8^ computed by our server.

To illustrate the usage, we introduce the details of the inputs (Figure 2) and outputs (Figure 3) with an example input: protein structure of the T4 lysozyme L99A/M102Q in complex with *N*-phenylglycinonitrile (PDB code: 2RBN).

**Figure 2:**
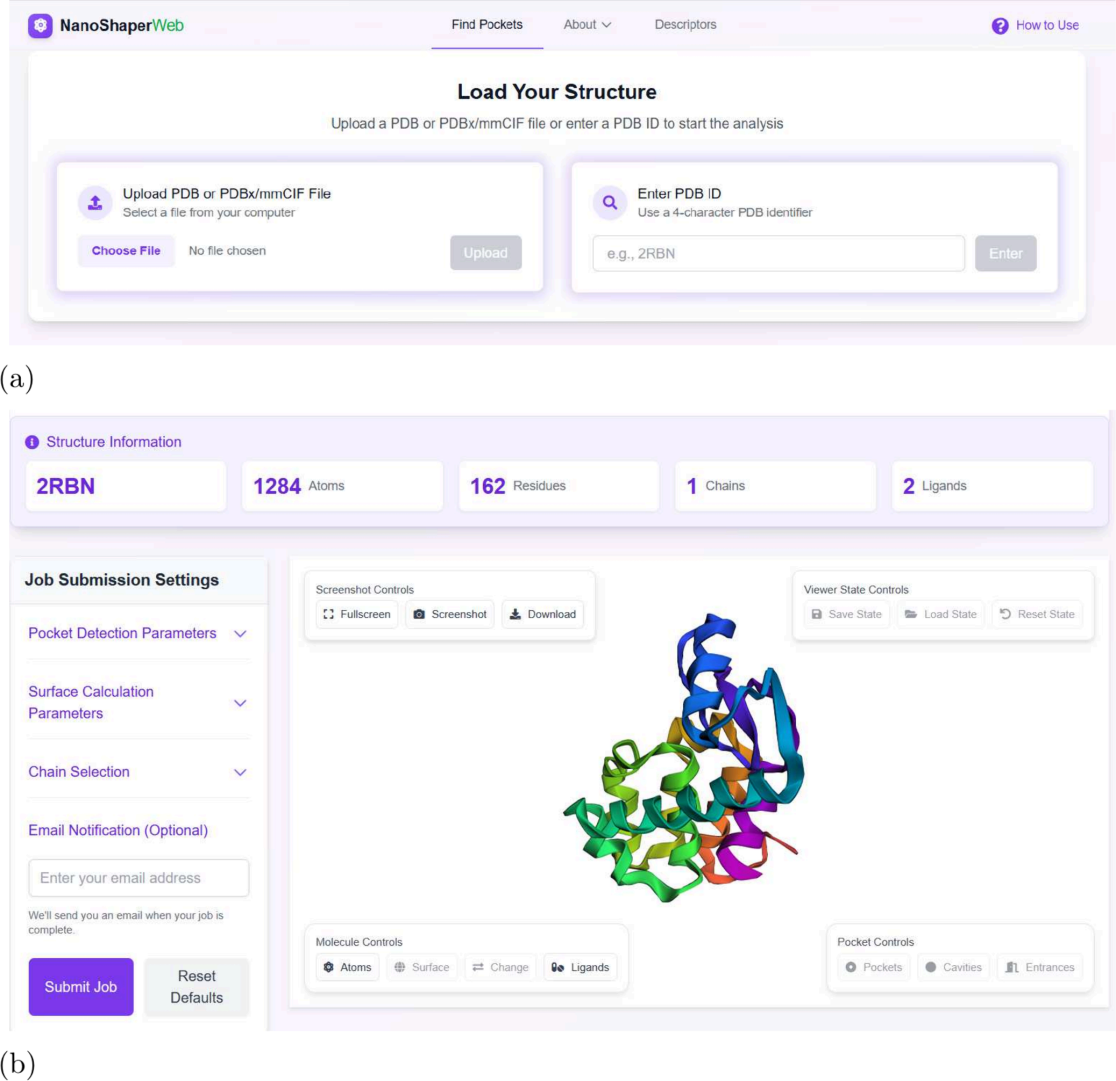
*Find Pockets* page and starting a new session. (a) In the Find Pockets page users can start a new job session. Here, users can specify the protein for analysis uploading or downloading the PDB of interest. As a representative example, the 2RBN protein is selected in this case. (b) The PDB file has been successfully downloaded and is ready for analysis. Key structural information is displayed, and users can interactively adjust the PDB visualization (e.g., atom-based views) to suit their needs. Clicking the “Submit Job” button on the left initiates the pocket detection process, placing the job in the Jobs queue with a unique job ID. The “Job Submission Settings” tab enables users to fine-tune various parameters for more precise control over the analysis.

**Figure 3:**
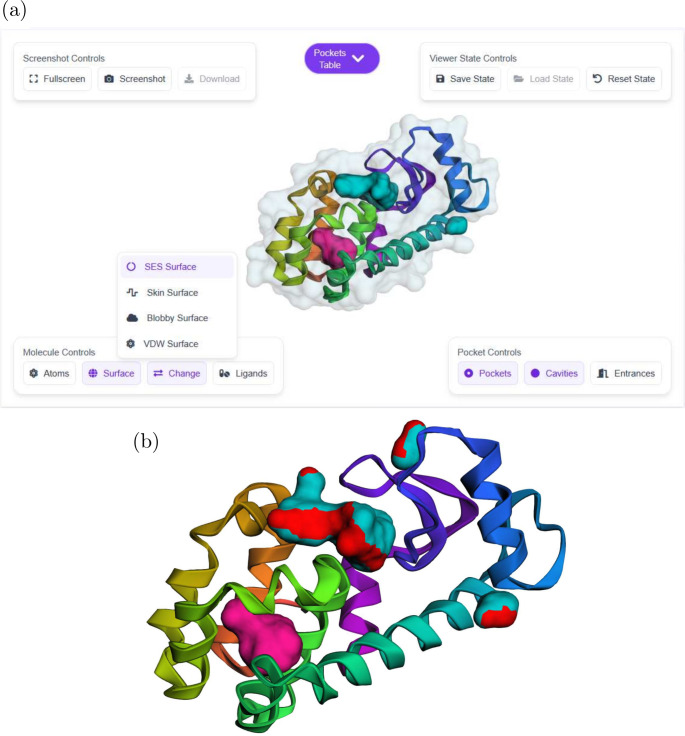
Output. (a) After the analysis, the protein can be visualized by four different surfaces: Connolly-Richards surface, skin surface, blobby surface, and VdW surface. By default, pockets and cavities are highlighted on the surface in distinct colors for easy identification. (b) Full screen screenshot of 2RBN protein.

### Input, Settings and Feed

In the *Find Pockets* page, users can choose to either start a new session or resume a previous analysis by providing the session ID. Selecting the “Start New Session” option reveals the input section where the protein file can be uploaded as a PDB/PDBx-mmCIF file or fetched directly from the web using the four characters PDB ID. Support for the new extended PDB identifier format is planned for future releases, ensuring continued compatibility as the PDB transitions to new standards.

After preprocessing, the system displays a molecular cartoon (Figure 2(b)) with structural details (residues, chains, atoms), customizable through visualization controls. Users initiate analysis by clicking “Submit Job,” with the resulting job appearing in the “Jobs Management” feed showing status, queue position, and action buttons. Advanced parameters (probe radii, skin smoothness, chain selection) are available in the Job Submission Settings tab.

For security, uploaded files are exclusively accessible to the user who uploaded them or those with whom the unique session ID is explicitly shared. All sessions and data are automatically deleted after 7 days.

### Viewer and Pocket Table

Once a job has completed, users can access the results by clicking the “View” button in the Jobs Management feed, which loads the results into the interactive viewer and pockets table components.

The molecular viewer is the central visualization component, surrounded by several control panels: (1) Molecule Controls for displaying atoms and surfaces, (2) Pocket Controls for toggling pockets and cavities visualization, including a special mode that highlights pocket entrances in red, distinct from pocket bodies (Figure 3(b)), which is particularly valuable for researchers seeking to identify potential access points to binding sites, (3) Viewer State Controls in the top right corner, providing tools for saving and restoring the entire viewer state (including all visibility settings, colors, and active pockets), facilitating consistent analysis across sessions and enabling researchers to return to specific visualization configurations, and (4) Screenshot Controls in the top left corner, offering functionality to capture high-resolution images of the current view, with options for full screen mode to maximize the visualization area before capture. The viewer supports standard 3D manipulation through mouse controls for rotation, panning, and zooming.

The pockets table provides an interface for managing pocket visualization and analysis. During the initial visualization, only the 10 largest pockets or cavities are loaded. Here, users have complete control over which pockets are displayed and how they appear. Each pocket entry includes essential metrics such as volume, surface area, type – i.e., cavity or pocket, with the ability to sort the table by any of these metrics by clicking the corresponding column header. Individual pockets can be added to or removed from the viewer using the dedicated button, and their colors can be customized through an integrated color picker. The pockets table supports efficient multiselection operations with Shift+click for selecting consecutive pockets and Ctrl+click for nonconsecutive selections. For detailed analysis, each pocket entry includes a “Show Details” button that reveals the entire set of descriptors calculated by the server. Changes made in the pockets table are immediately reflected in the viewer.

### Mail Notification and Results Download

NanoShaperWeb supports email notifications for job completion, with the optional email address specified in Job Submission Settings. Notifications contain session ID, job ID, and links to resume analysis or download results.

Results are available as ZIP archives containing:

- **Molecular Surface Files:** mesh representations of the computed surfaces in either MSMS or .off format, available for all four surface types. These files are viewable using VMD Software.
- **Individual Pocket/Cavity PDB Files:** separate PDB files for each detected pocket and cavity, facilitating targeted analysis of specific binding sites. These standard format files can be visualized with PyMOL, Chimera, or other molecular viewers.
- **Log Files:** a detailed log file containing a summary of key information from the analysis, including volume measurements, surface area calculations, and comprehensive lists of constituent atoms for all internal cavities and pockets identified in the molecular system.
- **Descriptor Data:** A CSV file containing the descriptors for each pocket, in a *machine learning-ready* format.

## Conclusions

The NanoShaperWeb application significantly enhances the accessibility of the NanoShaper framework by providing a web-based platform that requires no installation. This user-friendly interface allows researchers to easily perform molecular surface and pocket detection tasks, with computations handled remotely in parallel. The integration of DrugPred descriptors further enriches pocket detection, offering deeper insights into their features. By streamlining molecular analysis, NanoShaperWeb serves as an efficient and accessible tool for researchers, supporting crucial stages of the drug design process.

## Supplementary Material



## Data Availability

The Web server is available at https://nanoshaperweb.iit.it

## Notes

Additional details on the NanoShaper algorithm and the employed descriptors (PDF)
