# Supplementary material for: NanoShaperWeb: Molecular Surface and Pocket Detection Made Visual
Source: J Chem Inf Model. 2025 Jun 30;65(14):7341–6. doi: 10.1021/acs.jcim.5c00821 (PMC12308785; doi:10.1021/acs.jcim.5c00821)
Supplement: Supplementary file 1 [file ci5c00821_si_001.pdf]

Supplementary Information: NanoShaperWeb: molecular surface and pocket detection made visual

Carlo Abate,[*,†,‡,⊥] Eleonora Serra,[†,‡,⊥] Walter Rocchia,[¶] Andrea Cavalli,[§,‡] and Sergio Decherchi[*,‖]

†*Department of Pharmacy and Biotechnology (FaBiT), Alma Mater Studiorum - University of Bologna, via Belmeloro 6, 40126 Bologna, Italy*

‡*Computational & Chemical Biology, Fondazione Istituto Italiano di Tecnologia, via Morego 30, 16163 Genoa, Italy*

¶*Concept Lab, Fondazione Istituto Italiano di Tecnologia, via Morego 30, 16163 Genoa, Italy*

§*Centre Européen de Calcul Atomique et Moléculaire (CECAM), Ecole Polytechnique Fédérale de Lausanne, 1015 Lausanne, Switzerland*

‖*Data Science and Computation Facility, Fondazione Istituto Italiano di Tecnologia, via Morego 30, 16163 Genoa, Italy*

⊥*These authors contributed equally to the work.*

E-mail: carlo.abate@uit.no; sergio.decherchi@iit.it

Technical details of NanoShaper algorithms

Surface Computation

NanoShaper works by embedding the protein into a cubic regular grid and triangulating the molecular surface through ray-casting. Casting rays along grid edges allows to analytically sample the molecular surface and simultaneously recover the in/out information needed for triangulation.

Pocket Detection

In NanoShaper, the detection of pockets is obtained through the computation of the volumetric difference between two regions of space defined by two distinct Solvent-Excluded Surfaces (SESs). The two SESs are computed with different probe radii R and r where $R > r$. The volumetric difference of these SESs is computed over the grids, next a clean-up step is done to remove noisy grid points and lastly a flood-filling algorithm is run to isolate pockets.

The size of the probes influences the detection of the shape of the pockets: a larger radius R identifies shallow pockets, while a larger radius r smoothens the inner surface gaps. The default values are 3.0 Å for R and 1.4 Å for r, with the smaller radius approximating the size of a water molecule.

Pockets and cavities are distinct entities, with pockets having a well-defined body and entrance, while cavities lacking such structure.

Descriptors Builder

Each pocket identified by NanoShaper is characterized by geometric and chemical descriptors, along with the entrance area and points provided by NanoShaper.

Pockets properties include hydrogen-bond donor and acceptor surface areas (dsa_t and asa_t), calculated around polar atoms. The hydrophobic surface area (hsa_t) is derived by

subtracting the hydrogen-bond donor and acceptor areas from the total surface area. Relative descriptors (dsa_r, asa_r, and hsa_r) are computed by normalizing each by the total binding site surface area, with the relative polar surface area (psa_r) defined as the sum of dsa_r and asa_r.

The remaining descriptors, related to amino acid composition, are calculated based on the occurrence of different amino acid classes grouped by their physicochemical properties. Moreover, the occurrence of each amino acid of type (in_X) is reported as a descriptor, calculated as the sum of all surface areas surrounding the amino acid X. This activity is done through a Python script running on top of the NanoShaper executable.

Table 1: Complete list of descriptors and their corresponding names calculated by the NanoShaperWeb application.

**Descriptor**	**Name**
Binding site volume	vol
Total surface area	$area_b$
Entrance area	$area_e$
Binding site compactness	$cness$
Relative hydrogen-bond donor surface area	dsa_r
Hydrogen-bond donor surface area	dsa_t
Relative hydrogen-bond acceptor surface area	asa_r
Hydrogen-bond acceptor surface area	asa_t
Relative hydrophobic surface area	hsa_r
Hydrophobic surface area	hsa_t
Relative occurrence of polar amino acids	paa
Relative occurrence of non-polar amino acids	haa
Relative occurrence of multifunctional amino acids	maa
Relative occurrence of charged amino acids	caa
Relative polar surface area (dsa_r + asa_r)	psa_r
Incidence of amino acid X in the binding site relative to the surface	in_X
